# Wide-Bandgap Metal Halide Perovskites for Tandem Solar Cells

**DOI:** 10.1021/acsenergylett.0c02105

**Published:** 2020-12-23

**Authors:** Jinhui Tong, Qi Jiang, Fei Zhang, Seok Beom Kang, Dong Hoe Kim, Kai Zhu

**Affiliations:** † Chemistry and Nanoscience Center, National Renewable Energy Laboratory, Golden, Colorado 80401, United States; ‡ Department of Nanotechnology and Advanced Materials Engineering, Sejong University, Seoul 05006, Republic of Korea

## Abstract

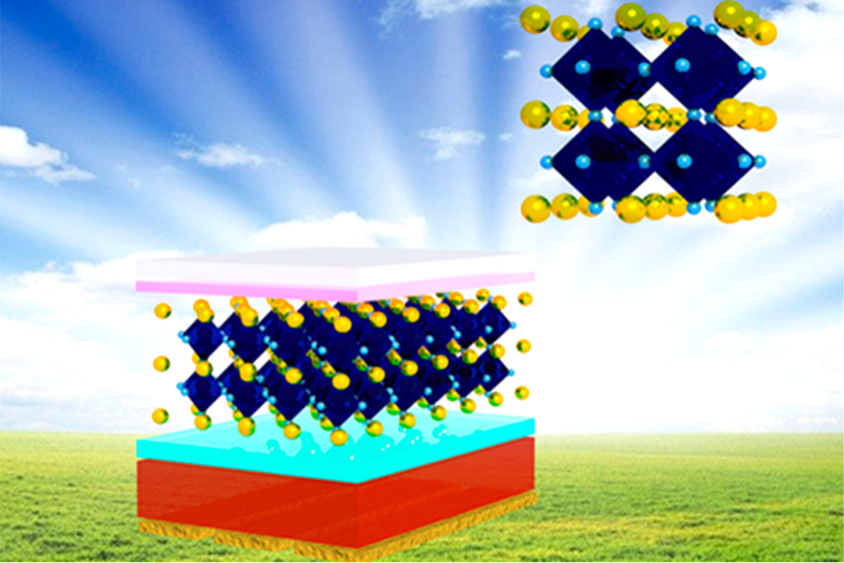

Metal halide perovskite solar cells (PSCs) have become the most promising new-generation solar cell technology. To date, perovskites also represent the only polycrystalline thin-film absorber technology that has enabled >20% efficiency for wide-bandgap solar cells, making wide-bandgap PSCs uniquely positioned to enable high-efficiency and low-cost tandem solar cell technologies by coupling wide-bandgap perovskites with low-bandgap absorbers. In this Focus Review, we highlight recent research progress on developing wide-bandgap PSCs, including the key mechanisms associated with efficiency loss and instability as well as strategies for overcoming these challenges. We also discuss recent accomplishments and research trends on using wide-bandgap PSCs in perovskite-based tandem configurations, including perovskite/perovskite, perovskite/Si, perovskite/CIGS, and other emerging tandem technologies.

Metal halide perovskite solar cells (PSCs) have made rapid development over the past decade.^1,2^ The attractive features of a perovskite absorber for photovoltaic (PV) applications include high absorption coefficient, low exciton binding energy, high carrier mobility, long carrier diffusion length, and ambipolar charge transport.^3−7^ With power conversion efficiency (PCE) climbing from 3.8% in 2009 to 25.5% in 2020, PSCs are the fastest-evolving PV technology today.^8−14^ At the current stage, although significant challenges still exist, perovskite PV holds promise for commercialization in the near future.^15^ Further increasing device efficiency represents an effective way to reduce the levelized cost of electricity to help increase the PV impact on the market, because the cost of module installation drops when the number of panels needed for installation to reach a desired power output is reduced.

A direct way for improving PV device efficiency is to build tandem solar cells. For single-junction solar cells, the ultimate PCE is governed by the Shockley–Queisser limit.^16^ The primary energy loss of a single-junction device includes the unabsorbed long-wavelength photons above the bandgap and thermalization of high-energy carriers.^17^ The tandem design can better harness solar energy: high-energy photons are absorbed by the upper wide-bandgap subcell, and long wavelength photons are absorbed by the bottom low-bandgap subcell.^18^ A key factor that makes perovskites highly attractive for tandem solar cells is their tunable bandgap by composition engineering.^19^ For example, by alloying iodide (I) with bromide (Br), the bandgap of lead (Pb)-halide perovskites can be continuously tuned from 1.47 to 2.3 eV. To date, perovskite is the only polycrystalline thin-film absorber that has demonstrated >20% PCE with a wide bandgap (around 1.7 eV or larger). This has made perovskites a highly relevant technology, not only for all-perovskite tandem but also for hybrid tandem architectures, where perovskite wide-bandgap devices are coupled with other mature low-bandgap PV technologies (e.g., silicon [Si] and copper indium gallium diselenide [CIGS] solar cells). The low-cost aspect of perovskite PV also ensures minimum additional cost when building perovskite-based tandem devices.

Wide-bandgap PSCs have already been successfully integrated with low-bandgap absorbers such as Si, CIGS, and Sn–Pb-based perovskites to make tandem solar cells with promising efficiencies.^20−29^Figure 1 provides an overview of the performance of various wide-bandgap PSCs used in perovskite/Si, perovskite/CIGS, and perovskite/perovskite tandem solar cells. It is clear that most efforts have focused on the 1.55–1.70 eV perovskites.^20,23−28,30−51^ Although the ideal bandgap for the top wide-bandgap PSC is close to 1.7 eV for Si or CIGS bottom cells, a higher bandgap around 1.8 eV is preferred to pair with the bottom low-bandgap PSC with a bandgap around 1.2–1.3 eV. It is also evident that significant room exists for improvement on all PV parameters including the short-circuit current density (*J*_sc_), open-circuit voltage (*V*_oc_), and fill factor.^52^ As a key component in all these perovskite-based tandem solar cells, especially for perovskite/Si and perovskite/CIGS tandem devices, the efficiency and stability of wide-bandgap perovskite subcells can limit the tandem device performance. Note that for a perovskite/perovskite tandem device, the development of low-bandgap Sn–Pb-based PSC is another key limiting factor on tandem performance, especially operational stability.^27,28,53^ Despite extensive studies on wide-bandgap PSCs during the past several years, there are still significant issues in both efficiency and stability. More research efforts are required on wide-bandgap PCSs to make highly efficient and stable perovskite-based tandem solar cells.

**Figure 1:**
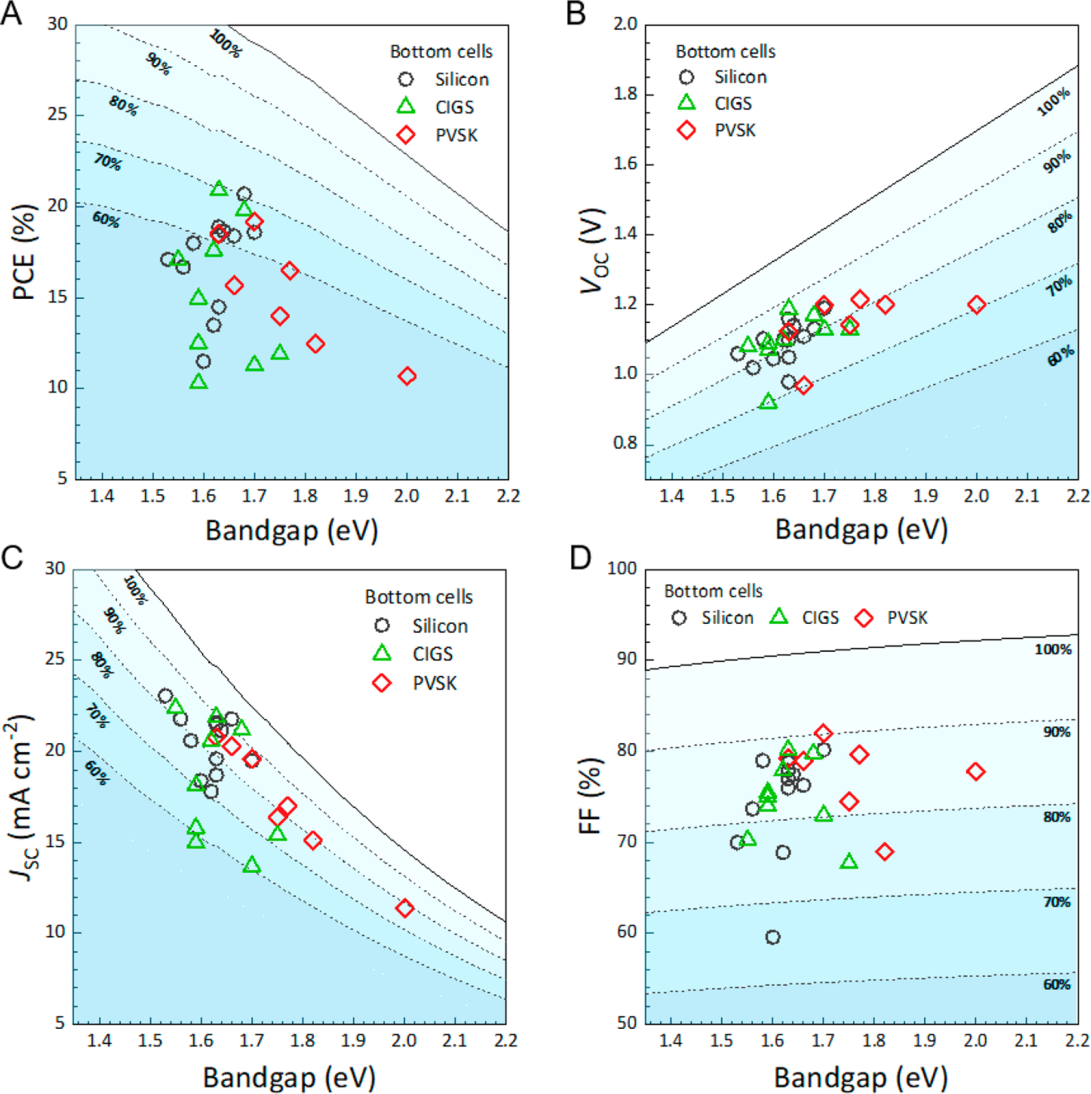
Reported photovoltaic parameters of wide-bandgap perovskite solar cells in the two-terminal tandem solar cells integrated with silicon (black circles), CIGS (green triangles), and perovskite (PVSK; red diamonds). The dotted lines in each panel indicate the relative values of the Shockley–Queisser limit.

In this Focus Review, we discuss recent research progress on developing wide-bandgap PSCs. We highlight strategies for improving the efficiency and stability of wide-bandgap PSCs. We focus on the mechanisms associated with voltage loss and phase segregation as well as the methodologies to address these challenges in wide-bandgap PSCs. We also highlight recent accomplishments and research trends on using wide-bandgap PSCs in perovskite-based tandem configurations with both two-terminal (2T) and four-terminal (4T) perovskite/perovskite, perovskite/Si, and perovskite/CIGS tandem technologies.

**Efficiency Improvement of Wide-Bandgap PSCs**. According to recent studies, the PCEs of perovskite-based tandem solar cells (including perovskite/Si, perovskite/CIGS, and perovskite/perovskite configurations) are expected to reach over 30%.^54,55^ To reach this target, it is necessary to develop highly efficient and stable wide-bandgap PSCs as a top subcell in these tandem configurations. For the wide-bandgap perovskite, the targeted bandgap is normally in the range of about 1.7 eV or higher to accommodate different bottom subcell technologies with a bandgap of about 1.1–1.3 eV. Increasing the halide ratio of Br/I in the perovskite composition is the most straightforward way to get a wider bandgap. Note that PSCs with a wider bandgap are not always accompanied by a higher *V*_oc_. Wide-bandgap PSCs usually suffer from large *V*_oc_ loss relative to their theoretical limit.^56−58^ Therefore, tremendous efforts have been devoted to reducing *V*_oc_ deficits in wide-bandgap PSCs.

*Additives Engineering*. Using additives in perovskite synthesis is a common method to boost the performance of wide-bandgap PSCs.^59^ The general functions of additives include improving the morphology of perovskite films, suppressing phase segregation, passivating traps in the bulk and/or at the surface of perovskite grains, and adjusting the interface energy level. As follows, several common additives such as two-dimensional (2D) materials and alkali metal cations are exemplified.

2D materials with large organic cations, such as phenylethylammonium (PEA^+^), butylammonium (BA^+^), and guanidinium (Gua^+^), are widely applied in 3D perovskites to improve device performance.^22,26,60−62^ Initially, the 2D Ruddlesden–Popper phase of layered perovskites was developed to overcome the stability issue of 3D perovskites.^63^ Compared with small cations (e.g., methylammonium [MA^+^] and formamidinium [FA^+^]) in 3D perovskites, the large, bulky cations in 2D perovskites can act as a spacer between the lead halide perovskite planes. These large, bulky 2D organic cations are usually hydrophobic, which can suppress moisture penetration. Thus, 2D perovskites usually exhibit better moisture stability compared with 3D perovskites. However, 2D PSCs generally exhibit relatively inferior efficiency compared with pure 3D PSCs, owing to their wide optical bandgap and suppressed charge transport in and between the 2D layers. To effectively balance the efficiency and stability, an alternative and effective approach is to use a small number of 2D perovskites as additives to mix into 3D perovskites to form 2D/3D hybrid structures.^64^

In 2017, Wang *et al*. showed that by carefully regulating the BA content in the 3D perovskite thin-film preparation, they obtained a unique heterostructure consisting of BA-based 2D platelets embedded between 3D perovskite grains (Figure 2A), aligned perpendicularly to the plane of the film.^60^ With this 2D/3D heterostructure, researchers observed greatly enhanced crystallinity, along with reduced defects responsible for suppressed nonradiative recombination. In addition, they also observed a slight blue shift of the photoluminescence (PL) emission, which was attributed to the shrinkage of the 3D perovskite lattice with BA incorporation. With these structural improvements, the efficiency of 1.72 eV wide-bandgap FA_0.83_Cs_0.17_Pb(I_0.6_Br_0.4_)_3_ was increased from 15.3% to 17.3% with reduced current density–voltage hysteresis.^60^

**Figure 2:**
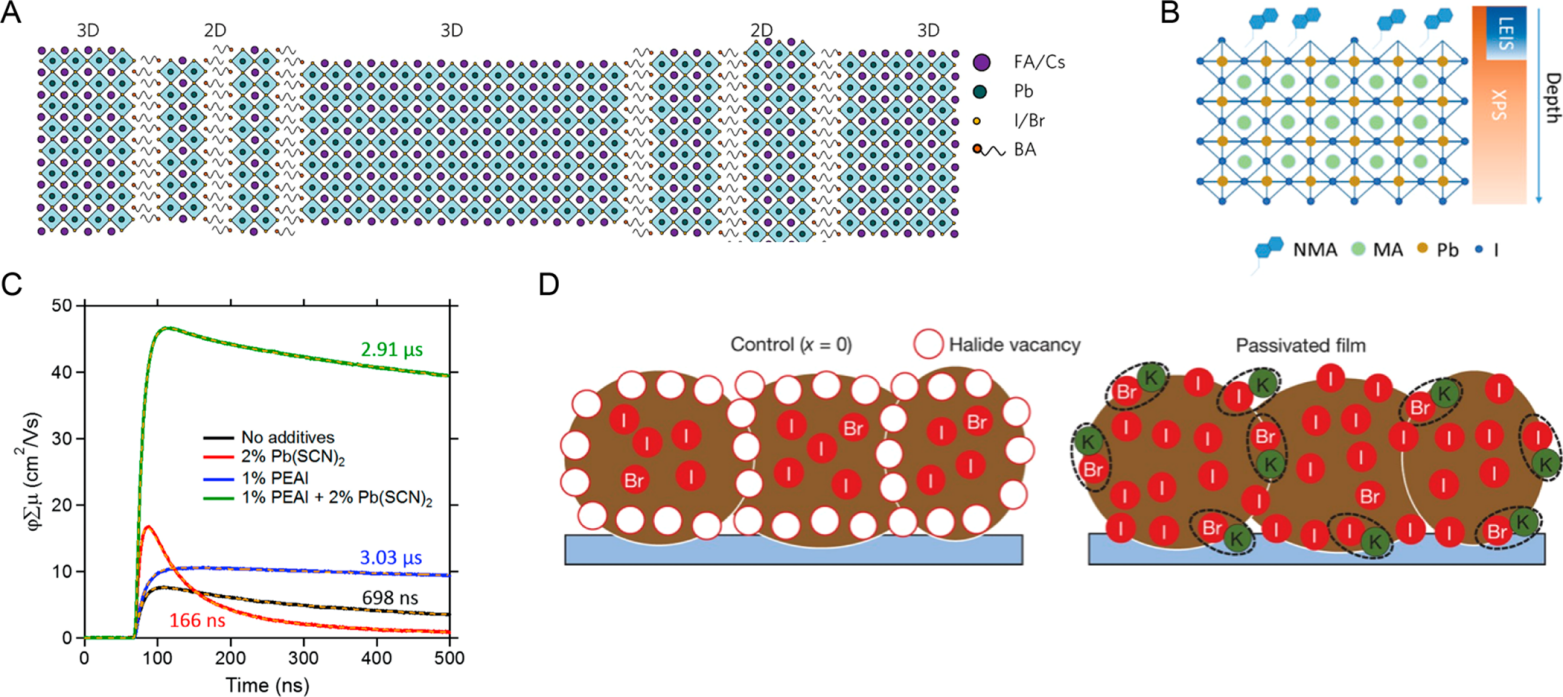
(A) Schematic illustration of the proposed self-assembled 2D/3D perovskite crystal interface. Reprinted with permission from ref (60). Copyright 2017 Nature Publishing Group. (B) Schematic of the X-ray photoelectron spectroscopy and low-energy ion scattering measurement. Reprinted with permission from ref (61). Copyright 2019 Wiley-VCH. (C) Charge-carrier dynamics of perovskite films with different additives measured by time-resolved microwave conductivity. Reprinted with permission from ref (37). Copyright 2019 Elsevier. (D) Schematic of surplus halide immobilized through complexing with K^+^ at grain boundaries and surfaces. Reprinted with permission from ref (69). Copyright 2018 Nature Publishing Group.

Lin *et al*. recently reported that the 2D bulky cation 1-naphthylmethylamine (NMA) could also suppress nonradiative recombination losses and enhance device efficiency in mixed iodide/bromide wide-bandgap PSCs.^61^ X-ray photoelectron spectroscopy and low-energy ion scattering measurements showed that NMA was partially covering on the grain surface, rather than being incorporated in the bulk of perovskite grains (Figure 2B). At low concentrations, NMA can passivate grain surface defects, whereas too much excess NMA can block charge transfer to the contact layers. High-quality perovskite layers with grains oriented perpendicular to the substrate are necessary to improve charge transport in 2D/3D PSCs; otherwise, the bulky cation may function as a barrier, inhibiting charge transfer. When NMA was applied in preparing 1.68 eV MAPb(I_0.8_Br_0.2_)_3_, the *V*_oc_ of the corresponding PSCs was significantly improved with an average increase from 1.13 to 1.21 V.^61^

Although the 2D additives could passivate traps, the large-size organic cations may inhibit charge transfer, which is not favorable for efficiency improvement. This challenge can be mitigated by increasing the grain size to reduce the number of grain boundaries and/or by engineering the 2D additives to improve charge transport through and within the 2D planes. In 2019, Kim *et al*. demonstrated an efficient bimolecular additives method to get highly efficient wide-bandgap (∼1.68 eV) PSCs.^37^ In this study, two complementary additives, phenylethylammonium iodide (PEAI) and Pb(SCN)_2_, were used to overcome the separate issues associated with each additive. Using the PEAI 2D bulky cation can passivate traps at perovskite grain boundaries; however, it may also increase the number of grain boundaries and impede charge transport. Using Pb(SCN)_2_ could enlarge the grain size but may form excess PbI_2_. The combination of PEAI and Pb(SCN)_2_ modulated the morphology, passivated traps, and reduced energy disorder, leading to markedly improved optical and electrical properties of the perovskite films with near 50 cm^2^ V^–1^s^–1^ mobility and 3 μs lifetime (Figure 2C).^37^ In another study, Ye *et al*. reported 2D bulky cation engineering by using a small amount of pentafluorophenethylammonium (F5PEA^+^) to partially replace PEA^+^ as a 2D perovskite passivation agent (2D-PPA), which forms a strong noncovalent interaction between the two bulky cations and improves charge transport by a factor of 3–5, leading to a demonstration of ∼21% 1.68 eV PSCs.^62^ Kim *et al*. further demonstrated that I^–^-SCN^–^ anion engineering of PEA^+^-based 2D additive was effective to facilitate charge transport across grain boundaries in 2D/3D perovskite films, resulting in a demonstration of ∼27%-efficient perovskite/Si 2T tandem devices.^26^ These studies demonstrate the importance of engineering 2D perovskite additives to control the optoelectrical properties.

Alkali metal cations are also useful candidates for defect passivation. Positively charged small-size alkali cations (e.g., Cs^+^, Rb^+^, Na^+^, and K^+^) can interact with the negatively charged defects in perovskites. Alkali cations were initially reported to improve the efficiency and stability of normal bandgap (1.5–1.6 eV) PSCs.^65−68^ Later, Stranks *et al*. showed that K^+^ could improve efficiency and stability across a wide range of bromide fractions, covering the ideal range of wide bandgaps (∼1.7–1.9 eV) for perovskite–perovskite tandem applications.^69^ They showed that K^+^ could decorate the perovskite surface and grain boundary to passivate defects (Figure 2D). In addition, potassium iodide (KI) could donate anion (I^–^) to fill I^–^ vacancies resulting from ion migration. With proper control of KI concentration, the devices based on a ∼1.8 eV (Cs,FA,MA)Pb(I_0.4_Br_0.6_)_3_ perovskite showed significant *V*_oc_ increase from 1.12 to 1.23 V, leading to a 17.5% efficient device, which represents a remarkable breakthrough in wide-bandgap PSC development.^69^

*Interfacial Engineering*. The interfaces between the perovskite layer and the electron- or hole-transporting layers are also important for wide-bandgap PSC performance. Dangling bonds on the surface, for example, methylammonium (MA) deficiency during thermal annealing of perovskite films, could act as traps affecting charge extraction.^70^ Ion migration and charge accumulation at the two interfaces may produce capacitive current, which accounts for one origin of hysteresis.^71^ In addition, nonmatched energy levels at these interfaces may lead to *V*_oc_ loss. In this section, we discuss the significance and approaches for interfacial engineering.

The concept of 2D/3D heterostructures can be applied to interfacial engineering. Depositing a 2D perovskite layer on top of the 3D perovskite surface could form a 2D/3D heterostructure. The 2D perovskite layer at the top surface has been shown to effectively block ion migration, remove defects, and suppress phase segregation of wide-bandgap PSCs.^22,72^ Benzylamine (BA) was found to passivate the defective regions and prevent the progression of decomposition or phase segregation in perovskite thin films through forming a 2D/3D heterostructure (Figure 3A).^72^ Post-treatment of 1.72 eV Cs_0.15_FA_0.85_Pb(I_0.73_Br_0.27_)_3_ perovskite thin film with a 2.5-vol % BA solution in chlorobenzene, followed by 10 min annealing at 140 °C, results in formation of some thin flake-like domains on the film surface, which corresponds to a 2D structure of the BA_2_PbI_4_ perovskite, as manifested by low-angle (6.2° and 7.3°) X-ray diffraction (XRD) peaks. The heterostructure of the top 2D perovskite layer and the underneath 3D perovskite created an energy-cascade structure, facilitating hole extraction while blocking electrons.^72^ In another study, Paetzold *et al*. reported BABr post-treatment on a 1.72 eV Cs_0.17_FA_0.83_Pb(I_0.6_Br_0.4_)_3_ perovskite, where a 2D interlayer of BA_*y*_(Cs_*x*_FA_1–*x*_)_1–*y*_Pb_2_(Br_0.4_I_0.6_)_7_ with *n* = 2 was formed between the absorber and hole transport layer (HTL).^73^ The wide-bandgap n-i-p PSCs with BABr treatment showed a *V*_oc_ of up to 1.31 V, representing the highest reported *V*_oc_-to-*E*_g_ ratio of 0.76 for wide-bandgap PSCs.

**Figure 3:**
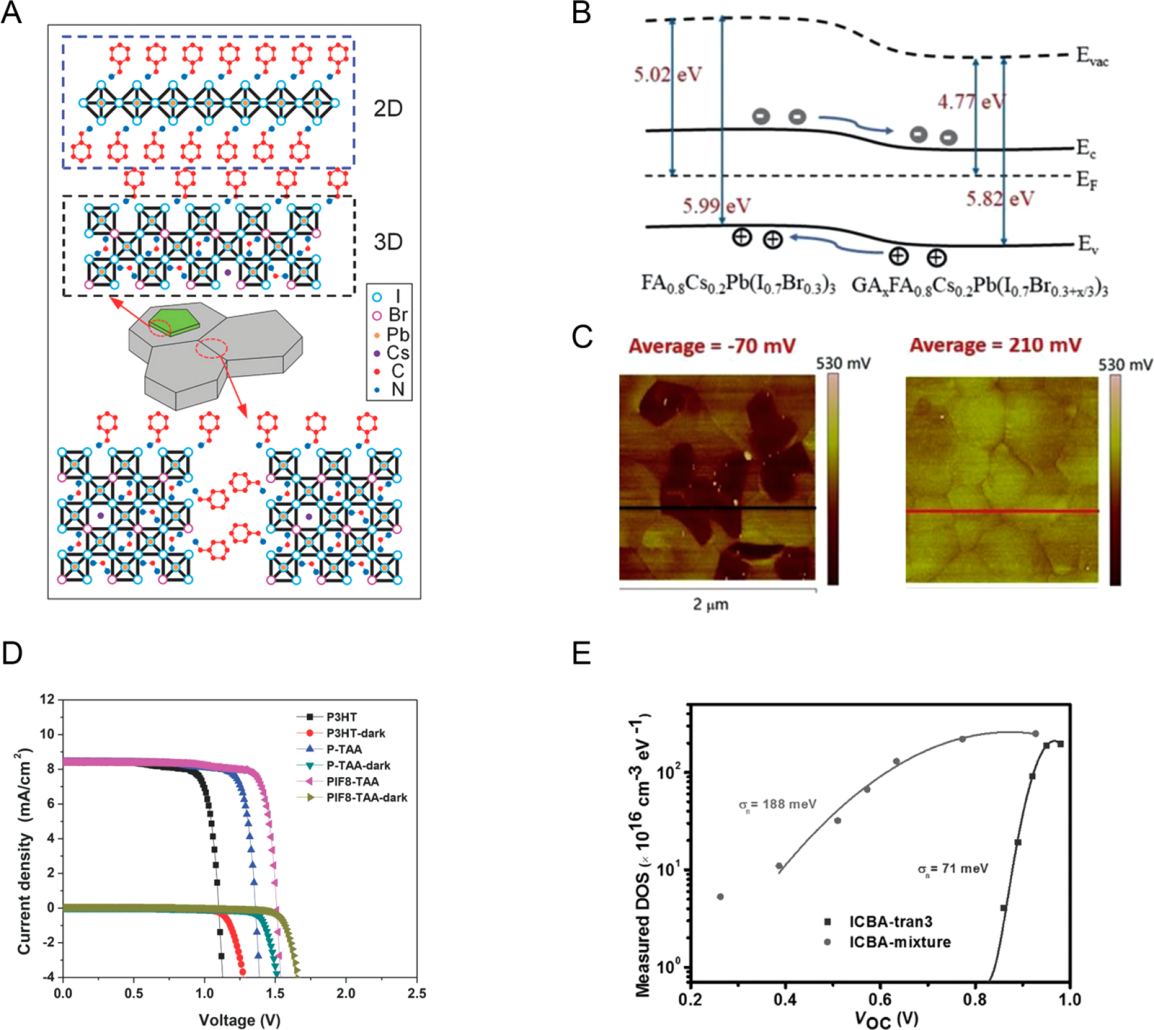
(A) Schematic diagram of 2D/3D interface at the 3D perovskite surface and grain boundaries. Reprinted with permission from ref (72). Copyright 2017 Wiley-VCH. (B) Schematic energy diagram of 3D perovskite treated with guanidinium bromide(GABr). (C) Kelvin probe force microscopy (KPFM)-measured surface potential of 3D perovskite film treated without (left) and with GABr (right). (B and C) Reprinted with permission from ref (74). Copyright 2019 Elsevier. (D) *J*–*V* curves of MAPbBr_3_ PSCs with different HTLs. Reprinted from ref (75). Copyright 2014 Wiley-VCH. (E) Measured density of states (DOS) of perovskite devices with ICBA-mixture or ICBA-tran3 extracted from impedance results. Reprinted from ref (76). Copyright 2017 Wiley-VCH.

Another way of interface engineering to achieve high device *V*_oc_ can also be achieved by effective doping and tuning the electronic properties of the surface of perovskite films. For instance, Yan *et al*. reported using guanidinium bromide (GABr) post-treatment to tune the electronic properties of the surface layer of 1.75 eV FA_0.8_Cs_0.2_Pb(I_0.7_Br_0.3_)_3_ perovskite thin film in p-i-n PSCs, via formation of a graded perovskite surface structure with the composition of GA_*x*_FA_0.8_Cs_0.2_Pb(I_0.7_Br_0.3+*x*/3_)_3_ (Figure 3B), to reduce the *V*_oc_ deficit.^74^ Kelvin probe force microscopy (KPFM) surface potential measurements showed that the working function increased by ∼280 meV with GABr treatment (Figure 3C), in agreement with the ultraviolet photoelectron spectroscopy results. The GABr treatment also converts excess PbI_2_ particles at grain boundaries to the perovskite phase, resulting in a more compositionally uniform perovskite surface. The combination of a compositionally uniform surface with a high surface potential facilitates charge separation at the perovskite/ETL interface, leading to increased *V*_oc_ from 1.12 to 1.24 V.^74^

Energy levels of the charge-transporting layers are another important factor that can limit the *V*_oc_ of PSCs. Wide-bandgap perovskites usually have a low valence band maximum (VBM) and high conduction band minimum (CBM), and consequently, large energy offsets can exist with commonly used HTLs and ETLs. Various HTLs, such as carbon nanotubes, conjugated polymers, nickel oxide, and spiro-OMeTAD, have been used in wide-bandgap PSCs. Notably, Heo *et al*. reported^75^ a record *V*_oc_ of 1.51 V for MAPbBr_3_-based PSCs by switching the HTL from P3HT (highest occupied molecular orbital [HOMO]: −5.0 eV) to PTAA (−5.14 eV) to PIF8-TAA (−5.51 eV), resulting in *V*_oc_ increased from 1.09 V (P3HT) to 1.35 V (PTAA) to 1.51 V (PIF8-TAA) (Figure 3D). Efforts on adjusting ETL energy levels were also conducted to improve *V*_oc_. Fullerene and its derivatives are the most widely used ETL in wide-bandgap PSCs, mainly for the following reasons: (1) the energy levels could be easily tuned; (2) fullerene and its derivatives could function as both ETL and passivation agents at the surface and grain boundaries of the perovskite films; and (3) the processing of fullerene and its derivatives is usually compatible with the underlying perovskite layers.^76^ Among various fullerene and fullerene derivatives, [6,6]-phenyl-C61-butyric acid methyl ester (PCBM) and C60 are the two most commonly used ETLs. Indene-C60 bis-adduct (ICBA) was shown to be better than PCBM for wide-bandgap PSCs because of its higher lowest unoccupied molecular orbital (LUMO) level (−3.7 eV) than PCBM (−3.9 eV).^76^ A higher LUMO elevates the quasi-Fermi level of electrons in the ETL and increases the *V*_oc_. However, ICBA is not easily crystallized compared with PCBM. The energy disorder of ICBA also reduces the upper limit of the quasi-Fermi level splitting and limits the maximum *V*_oc_. Lin *et al*. showed that isolating ICBA multiadducts is an efficient route to reduce its energy disorder (Figure 3E).^76^ ICBA-tran3 isolated from an ICBA-mixture has the same high LUMO level, but with much reduced energy disorder and higher conductivity, which results in ∼60 mV increase in *V*_oc_ for 1.71 eV (FA_0.83_MA_0.17_)_0.95_Cs_0.05_Pb(I_0.6_Br_0.4_)_3_-based PSCs, corresponding to a *V*_oc_ deficit of ∼0.5 V. It is noteworthy that a recent report recommends an approach to estimate the *V*_oc_ deficit value based on external quantum efficiency (EQE) spectra to determine the photovoltaic bandgap, rather than the commonly used optical bandgap.^77^

These above discussions clearly point to a few directions for making highly efficient wide-bandgap PSCs. A high-efficiency wide-bandgap PSC should first have a high-quality perovskite absorber having good crystallinity, low trap density, effective charge transport, and suppressed phase segregation. The surface and grain boundaries often possess various trap centers and thus should be passivated without affecting charge transport across boundaries or surfaces. Effective doping and tuning the electronic properties of the interface of perovskite films are usually necessary. Several approaches are effective, such as the design of 2D/3D heterojunction, graded perovskite surface structure with modified surface potential, and ETLs/HTLs with less energy disorder ETL and HTL.

**Stability Improvement of Wide-Bandgap PSCs**. A main challenge for PSCs or related tandem devices to be commercialized is the lack of demonstration of long-term (*e.g.*, 20 years) outdoor operation stability. During the past several years, significant advances have been made in understanding of degradation mechanisms and improving device stability. In this section, we discuss the common degradation factors for wide-bandgap PSCs as well as recent approaches to improve their stabilities.

*Degradation Factors*. Wide-bandgap PSCs share virtually all the instability characteristics that normal-bandgap PSCs possess. The degradation often results from chemical and structural changes of the perovskite active layers and can be accelerated by various factors, including moisture, light illumination, high temperature, and oxygen exposure. The structural stability of a 3D perovskite structure can be first estimated by the Goldschmidt tolerance factor, *t*, as given by *t* =  where *r*_A_, *r*_B_, and *r*_X_ are the radii of monovalent cation, divalent metal cation, and monovalent halide anion cation, respectively. The value of *t* should generally be in a certain range (0.8–1) to maintain the perovskite crystal structure. If *t* is less than 0.71 or greater than 1, nonperovskite structure can form. The organic–inorganic hybrid halide perovskites tend to form a hexagonal structure for *t* > 1, orthorhombic for *t* < 0.8, and cubic for 0.8 < *t* < 1. There is a general trend of replacing MA with FA in most perovskite compositions.^78^ Small cations (*e.g.*, cesium [Cs] and rubidium) have been alloyed with FA or MA in various perovskites to increase their quality, such as crystallinity and morphology, which often, in turn, affect perovskite stability.^66,79−82^

The presence of moisture and oxygen also can accelerate the degradation process for wide-bandgap PSCs, the same as for other PSCs. Even though oxygen may not cause harm on the device stability if stored in dark and dry conditions, the photo-oxidation-induced degradation is reported in PSCs.^83^ Through first-principles calculations, the degradation caused by the photo-oxidation was explained with three steps: generation of superoxide PbO on the perovskite surface under light, fast oxidation of the perovskite surface, and slow hydration of the perovskite inner regions.^83^ Additionally, the commonly adopted ETL and/or HTL employed for many wide-bandgap PSCs (same as normal-bandgap PSCs) were less stable in the oxygen-containing environment. For example, the organic HTL poly(triarylamine) (PTAA) may oxidize if exposed to air or O_2_, leading to degradation of devices.^84^ Moisture is known to be detrimental to the crystalline structures of perovskites and, consequently, a major instability factor for PSC operation.^85,86^ The ground- and excited-state absorption spectra of perovskites could be changed by moisture.^87^ In addition to affecting the perovskite absorber, moisture can also degrade HTL and ETL.^88^ Thus, strategies have been explored to improve the oxygen and moisture stability of PSCs. For example, a thin layer of aluminum zinc oxide (AZO), SnO_2_, or SnO_2_/zinc tin oxide (ZTO), by atomic layer deposition (ALD), as the ETL has been shown to effectively improve the thermal and environmental stability of PSCs.^20,89,90^ The dense and pinhole-free ALD-coated metal oxide layer can act as a diffusion barrier against oxygen/moisture penetrating into the perovskite active layers. By using an ALD SnO_2_/ZTO bilayer structure as the ETL, CsFA-based, wide-bandgap PSCs without encapsulation showed minimum degradation over 1000 h under continuous ∼1 sun illumination at about 40% relative humidity and ∼35 °C under lamp heating.^20^ Devices using inorganic HTLs, such as CuSCN or NiO_*x*_ instead of PTAA or spiro-OMeTAD, have also shown improved device stability.^91,92^

In addition to the above-mentioned instability issues commonly observed for most perovskite compositions, wide-bandgap PSCs often suffer from the halide-related phase segregation issue.^57,93,94^ Normally, wide-bandgap perovskites are created by partially replacing I^–^ with Br^–^ on the X anion site of the general perovskite structure, ABX_3_. The bandgap increases with a higher bromide ratio in AB(I_1–*x*_Br_*x*_)_3_.^94,95^ However, devices do not always exhibit higher *V*_oc_ with increasing bandgap.^58^ When the mixed-halide perovskite has >20% bromide, the device shows a decrease of *V*_oc_ with increasing Br^–^.^96^ This abnormal *V*_oc_ loss is often attributed to I^–^ and Br^–^ phase segregation.^93^ Hoke *et al*. initially studied the changes of PL and absorption spectrum of MAPb(Br_*x*_I_1–*x*_)_3_ under continuous light illumination (Figure 4A).^93^ They found that for perovskites with *x* > 0.2 for Br^–^, upon illumination, the initial PL intensity slowly decreased, and a new energy peak of lower energy developed with a much higher intensity, resulting from halide-phase segregation, forming narrow-bandgap, I-rich and wide-bandgap Br-rich regions. Photogenerated carriers can thus be trapped by the I-rich region, owing to the energy offset (Figure 4B). This phase segregation is reversible as the initial PL spectrum is recovered after removal of illumination.^93^ Many groups followed this initial study to further investigate halide-phase segregation in wide-bandgap perovskites.^57,97−100^ Sadhanala *et al*. reported similar halide-phase segregation even under inert, dark conditions over 21 days.^100^ This observation suggests a gradual and spontaneous phase segregation in mixed-halide, wide-bandgap perovskites. Light illumination can accelerate phase segregation by providing energy to assist halide-ion migration over certain energetic barriers. Bischak *et al*. further examined halide-phase segregation, combining multiscale modeling (Figure 4C) and nanoscale imaging (Figure 4D–F).^57^ They found that hybrid perovskites have a high static dielectric constant, resulting in strong electron–phonon coupling and large polarizabilities, and the exciton binding energy is small. As a result, the photogenerated electron–hole pairs can quickly dissociate into free electrons and holes, which, in turn, deform the surrounding perovskite lattice. For MAPb(Br_*x*_I_1–*x*_)_3_, the lattice distortion increases enthalpy, affecting the free energy with perovskite composition, which provides a theoretical basis for the phase segregation phenomenon in mixed-halide perovskites.

**Figure 4:**
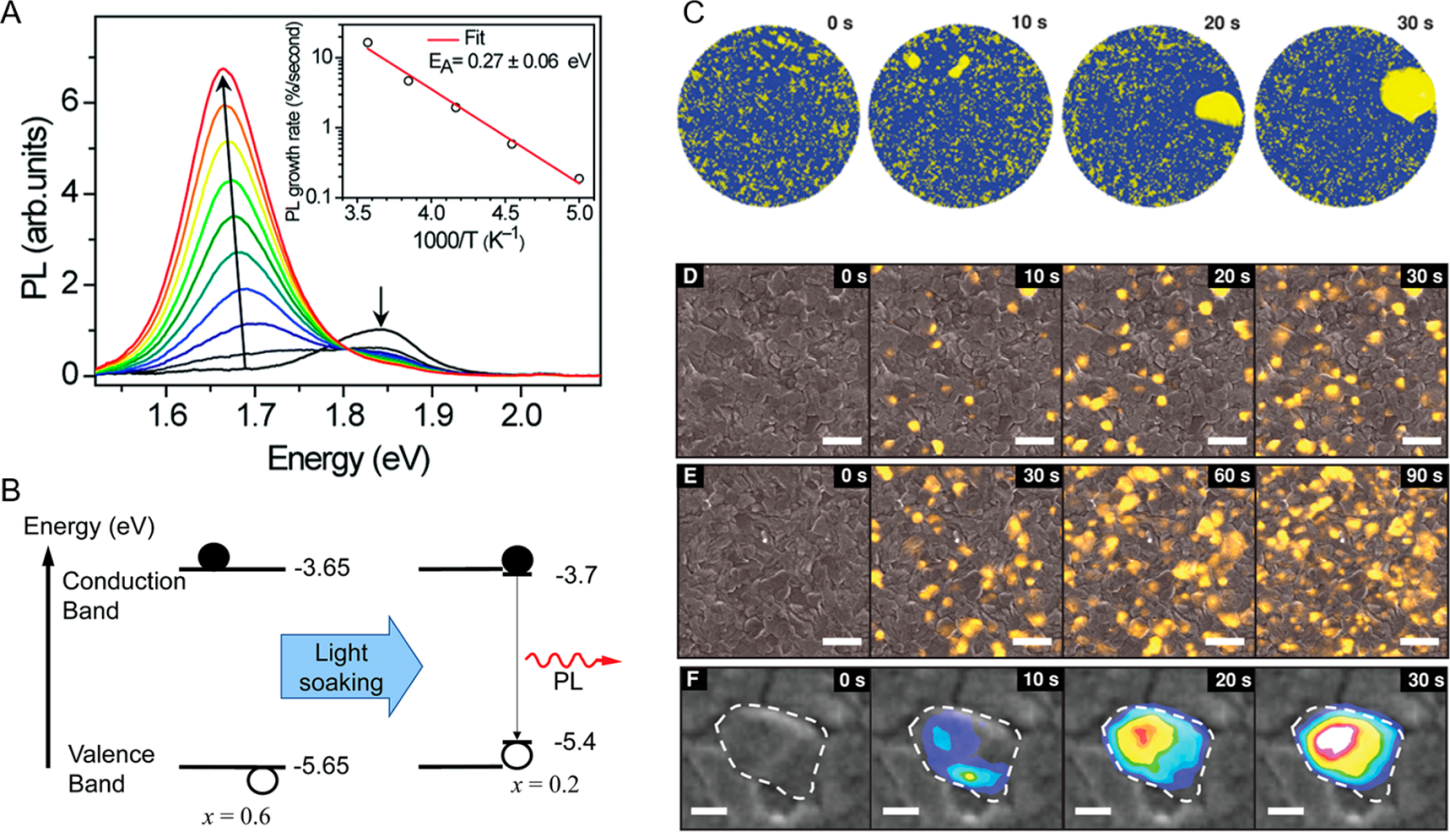
(A) PL of MAPb(Br_0.4_I_0.6_)_3_ thin film under illumination at 300 K. Inset shows the temperature dependence of the PL growth rate. (B) Schematic illustration of the photoinduced trap formation in mixed-halide perovskite film. (A and B) Reprinted with permission from ref (93). Copyright 2015 The Royal Society of Chemistry. (C) A series of snapshots from a domain-formation simulation with iodide-rich regions in yellow- and bromide-rich regions in blue. (D*–*F) Cathodoluminescence imaging of the formation and evolution of iodide-rich clusters. (C*–*F) Reprinted with permission from ref (57). Copyright 2017 American Chemical Society.

*Strategies for Stability Improvement*. Halide-based phase segregation in wide-bandgap perovskites is a result of separate I and Br diffusions within the perovskite films. Halide migration in perovskites is thought to occur through halogen vacancies, and ion conduction in a perovskite film is expected to be easier via grain boundaries.^101,102^ Thus, various strategies, *e.g.*, reducing defect density, enlarging the grain size, and reducing the number of grain boundaries, have been explored to mitigate halide migration.^103−105^ Additives are commonly used to modify the crystallization and improve film quality. Zhou *et al*. illustrated how precursor additive Pb(SCN)_2_ should be matched with a proper ratio of FAX (X: I and Br) to produce large grains with defect-healed grain boundaries.^105^ With this approach, the optimized wide-bandgap, FA_0.17_Cs_0.83_PbI_3–*x*_Br_*x*_, showed good photostability at room temperature and at 85 °C. Alkali cations were also useful to enhance the phase stability of wide-bandgap PSCs.^105^ For example, potassium ions have been experimentally shown to occupy interstitial sites in the perovskite lattice and form 2D K_2_PbI_4_ at the grain boundaries, both of which can passivate traps and prohibit ion migration, thus suppressing photoinduced phase segregation.^69^ In a device stack, the selection of HTL or ETL on the substrate also plays a significant role, affecting the morphology of the wide-bandgap perovskite deposited on top of HTL/ETL.^106^ Hu *et al*. showed that the MAPbBr_0.8_I_2.2_ grains formed on PTAA were much larger than those formed on PEDOT:PSS.^106^ The formation of the large grains on PTAA is ascribed to the hydrophobic nature of the PTAA surface, which is critical to perovskite nucleation and grain growth.

Bischak *et al*. reported that modifying perovskite composition could reduce the lattice distortion caused by photoexcited carriers.^57^ Reducing lattice distortions should facilitate uniform phase formation and help inhibit phase segregation. McMeekin *et al*. partially substituted FA for Cs and pushed the region of structural instability in the Br/I phase space to higher energies, thus achieving a structurally stable mixed-halide perovskite with a band gap of 1.75 eV.^107^ The phase-stable FA_0.83_Cs_0.17_Pb(I_1–*x*_Br_*x*_)_3_ perovskite was also confirmed by PL and XRD studies. B-site tuning of ABX_3_ wide-bandgap perovskites was also found as a potential approach to improve perovskite stability. Yang *et al*. successfully demonstrated stabilization of the I/Br phase by partially replacing Pb^2+^ with Sn^2+^ and verified this stabilization with XRD and transient absorption spectroscopy.^108^ They found increased micro strain and decreased crystal size under illumination for MAPb(I_0.6_Br_0.4_)_3_ as a result of compositional inhomogeneity due to phase segregation, whereas no such increase was observed for MAPb_0.75_Sn_0.25_(I_0.6_Br_0.4_)_3_. This phase-stability improvement was attributed to the partial replacement of Pb with Sn, which alters the driving force for phase segregation and increases the barrier for ionic diffusion associated with the hindrance from the added strain field and increased crystal size.^108^

All-inorganic perovskites without a volatile organic component have also attracted significant attention as a promising structure with good thermal stability for wide-bandgap PSCs.^109,110^ Cs is the most widely used A-site cation in all-inorganic perovskites, such as CsPbI_3_, CsPbI_2_Br, CsPbIBr_2_, and CsPbBr_3_. Among these compositions, CsPbBr_3_ has the highest stability; however, its bandgap, ∼2.3 eV, is too large for solar cell applications. CsPbIBr_2_ has a slightly narrower bandgap of ∼2.05 eV and still has good stability, but the efficiency of CsPbIBr_2_-based PSCs is currently limited to ∼10%.^111^ Recently, CsPbI_2_Br-based PSCs have seen rapid PCE improvement following efforts to enhance the crystallinity and reduce trap density; the best reported CsPbI_2_Br PSC has a *V*_oc_ of 1.32 V and a PCE of 16.79%.^112^ CsPbI_3_ is so far the only all-inorganic perovskite composition that has already shown a PCE > 19%.^113^ The bandgap of CsPbI_3_, ∼1.73 eV, is also promising for making tandem devices with other low-bandgap PV absorbers (*e.g.*, Si and CIGS). Moreover, the issue of halide inhomogeneity/segregation is absent for CsPbI_3_. However, the small-size Cs^+^ in CsPbI_3_ gives rise to a nonideal tolerance factor, causing phase instability under ambient conditions and changing CsPbI_3_ from perovskite α-phase to a nonperovskite orthorhombic phase (also referred to as the yellow phase or δ-CsPbI_3_) with poor optoelectronic properties.^114^ To overcome this issue, many efforts toward stabilizing α-CsPbI_3_ have been reported.^109,111,115^ Zhang *et al*. reported a phase-stable α-CsPbI_3_ film, even at temperatures several hundred Celsius below the phase transition point, by using a small amount of 2D EDAPbI_4_ (EDA: ethylenediamine).^116^ Theoretically, tetragonal (β-CsPbI_3_) polymorph CsPbI_3_ can be crystallized at lower temperatures and would have a more stable perovskite structure than the cubic α-CsPbI_3_.^116^ However, it is challenging to deposit stabilized β-CsPbI_3_. Recently, Wang *et al*. reported a stable β-CsPbI_3_ with a bandgap of 1.68 eV, enabled by using a dimethylammonium iodide (DMAI) additive and choline iodine (CHI) interface engineering, producing a PSC with 18.4% efficiency.^109^ The efficiency was further improved to 19.03% by optimizing DMAI additive content, coupled with using phenyltrimethylammonium chloride passivation treatment.^113^

Another promising approach toward stable wide-bandgap PSCs is to synthesize perovskites in the form of nanometer-sized quantum dots (QDs).^117−120^ Advantages of perovskite QDs include possible multiexciton generation, near-unity PL quantum yield, and additional bandgap tunability by the quantum-confinement effect.^121,122^ Perovskite QDs could have suitable wide bandgaps for tandem solar cells. For example, CsPbI_3_ QDs have a tunable bandgap from 1.75 to 2.13 eV.^117^ More importantly, both colloidal CsPbI_3_ and FAPbI_3_ QDs have shown improved phase stability at room temperature compared with their bulk thin-film counterparts.^117,119^ In comparison to the 3D bulk counterpart, QDs have been reported to retain the cubic phase because of the large contribution of surface energy.^117^ Perovskite QD research was initially centered on CsPbX_3_, which often exhibits improved room-temperature α-phase stability and attractive optical properties. Swarnkar *et al*. demonstrated α-CsPbI_3_-based QD solar cells with a high *V*_oc_ of 1.23 eV and phase stability for months in ambient air.^117^ Charge transport within the perovskite QD film is often a limiting factor to obtaining highly efficient perovskite QD solar cells. Sanehira *et al*. showed that charge mobility within the perovskite QD films is governed by the chemical conditions at the QD-to-QD junctions.^123^ They found that post-treatment perovskite QD films with AX (A: FA, MA, or Cs; X: I or Br) was effective to tune the coupling between QDs for enhanced charge transport. In addition to the CsPbI_3_QD solar cells, Hao *et al*. have recently reported mixed-Cs-FA-based Cs_1–*x*_FA_*x*_PbI_3_ QDs solar cells with significant advancement on efficiency and stability.^119^ In this study, the oleic-acid (OA), ligand-assisted cation-exchange method was used to synthesize Cs_1–*x*_FA_*x*_PbI_3_ QDs with a controlled Cs-FA mixing ratio, *x*, from 0 to 1. In the OA-rich environment, the cross-exchange of cations was enhanced, enabling rapid formation of Cs_1–*x*_FA_*x*_PbI_3_ QDs with high quality. With a composition of Cs_0.5_FA_0.5_PbI_3_, the QD film showed a bandgap of 1.64 eV with the corresponding device of 16.6% efficiency. These devices also exhibited substantially enhanced photostability, retaining 94% of the initial PCE under continuous 1 sun illumination for 600 h.^119^

**Perovskite-Enabled Tandem Solar Cells**. Tandem solar cells have the potential to overcome the Shockley–Queisser limit of single-junction solar cells.^18,55^ A single-junction solar cell with a particular bandgap can only absorb photons with energies higher than its bandgap. The excess energy above the bandgap is lost through the thermalization process. By stacking absorbers with different bandgaps in a tandem configuration, the high-energy photons are captured by the large-bandgap absorbers, while the low-energy photons are allowed to pass through for absorption by a low-bandgap subcell, enabling an efficient use of a wide range of photon energies. At present, the most efficient tandem devices are from III–V-based semiconductors with efficiencies up to ∼46% under concentrated sunlight.^124^ However, these tandem solar cells have expensive manufacturing costs, limiting their terrestrial applications. PSCs hold great promise in addressing the long-term bottleneck in obtaining low-cost and highly efficient tandem solar cells.^19^ PSCs with a wide bandgap around 1.7 eV or higher are the only polycrystalline thin-film PV technology to achieve a PCE around 20% or higher, which makes wide-bandgap PSCs uniquely suitable for enabling low-cost and highly efficient hybrid tandem configurations (perovskite/Si and perovskite/CIGS), along with the possibility for all-perovskite tandem devices. Note that for Si or CIGS bottom cells with a bandgap near 1.1 eV, the ideal bandgap of the perovskite top cell is close to 1.7 eV, and the corresponding perovskite composition is often based on the (CsFA)Pb(IBr)_3_ or (CsMAFA)Pb(IBr)_3_ with different mixing ratios on the A-site and X-site. For the low-bandgap perovskite bottom cell, which usually has a bandgap of ∼1.2–1.3 eV, the bandgap for the perovskite top cell is often increased to about 1.75–1.8 eV using the above-mentioned CsFA- or CsMAFA-based perovskite compositions. Research advances on wide-bandgap PSC development with both efficiency and stability improvements are discussed in detail in the previous two sections.

Dual-junction tandem solar cells are generally constructed in two configurations: 2T monolithic integration of two subcells and 4T mechanical stacking of two independent subcells. Each tandem structure has its own advantages and disadvantages. The 2T configuration has subcells in series connection through an interconnection layer (often referred to as the recombination layer). There are only two electrodes in a 2T tandem device, similar to a single-junction solar cell. Because the subcells are series-connected, the *V*_oc_ of the 2T tandem device is the sum *V*_oc_ of the two subcells, and the *J*_sc_ is limited by a subcell with the smaller *J*_sc_ (which is referred to as the current matching). In addition to the current matching requirement, it is often challenging to construct a reliable and efficient interconnection layer, which is critical to ensuring process compatibility among subcells and minimum optical and electrical losses during tandem operation. For a 4T tandem configuration, each subcell is separately connected to the external circuit. All subcells are fabricated independently, and the efficiency of a 4T tandem PSC is the sum from each subcell. Although it is easier to fabricate 4T tandem cells, each subcell is fabricated on a separated substrate with two electrodes to operate. Thus, a 4T tandem device is usually more costly to build and requires more external circuit controls for operation. In addition, the more electrodes involved, the more optical and electrical losses are likely to occur. Thus, most research efforts on perovskite-based tandem solar cells have focused on 2T configurations, including perovskite/Si, perovskite/CIGS, and perovskite/perovskite absorber technologies. Figure 5 shows the efficiency progress of these major perovskite-based 2T tandem devices.

**Figure 5:**
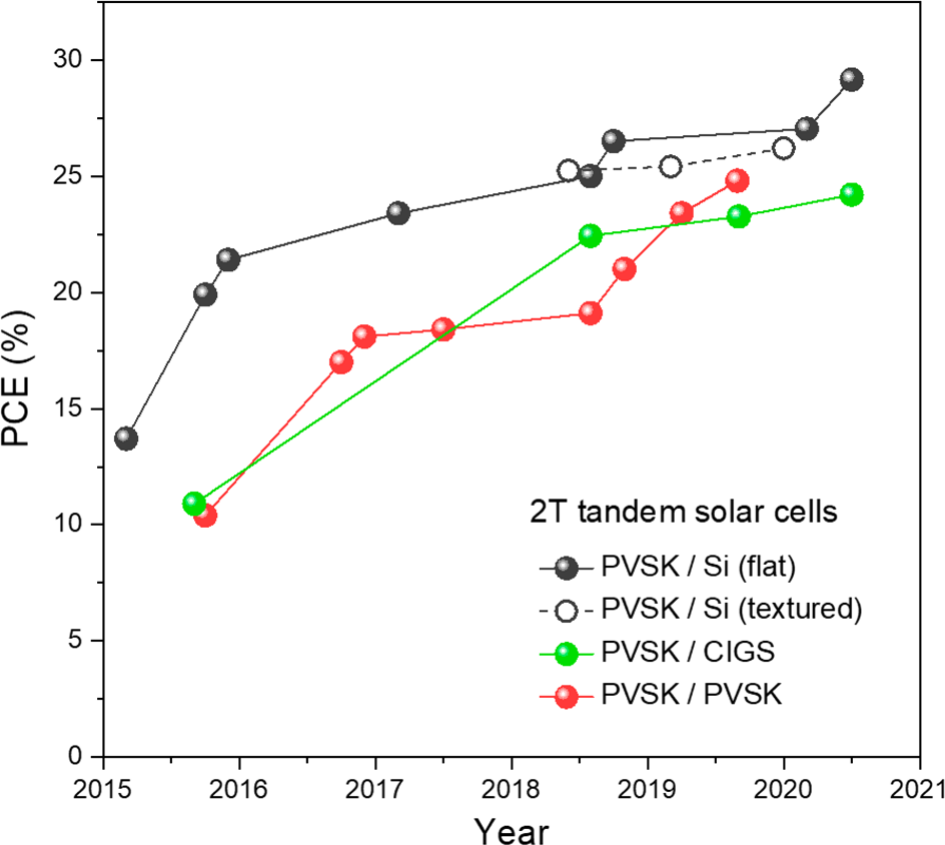
Efficiency evolution of perovskite-based 2T monolithic tandem solar cells, including perovskite/Si tandems with both flat and textured Si subcells, perovskite/CIGS tandems, and perovskite/perovskite tandems. PVSK: perovskite.

Fabricating highly efficient 2T tandem devices requires the successful preparation of all key device components: (1) wide-bandgap and low-bandgap subcells with proper pairing for efficient light harvesting and current matching; (2) transparent and conductive top electrodes to allow maximum transmission of the incident light with a minimum reflective loss; and (3) interconnection layer with minimum optical loss, lateral charge conduction, and maximum vertical charge conduction and protection of the underlying device layers.

*Perovskite/Si Tandem*. The first challenge to integrate a wide-bandgap perovskite subcell with a Si subcell is the development of highly transparent and conductive electrodes for the perovskite top cells, as the incident light needs to first go through the top perovskite subcell before entering the bottom Si subcell. The transparent electrodes should be highly transparent and conductive to minimize optical and electrical losses. Several types of semitransparent electrodes have been studied for semitransparent PSCs, such as silver nanowire mesh, graphene, carbon nanotubes, poly(3,4-ethylenedioxythiophene)-poly(styrenesulfonate) (PEDOT:PSS), and transparent conductive oxides (TCOs).^125−128^ After several years of active research, many groups have settled on the TCO approach, often based on sputtered indium-doped tin oxide (ITO) or indium-doped zinc oxide (IZO).^20,26,30,50,103^ In perovskite/Si tandem solar cells, the wide-bandgap perovskite subcell often uses an inverted PSC architecture, which is also referred to as the p-i-n structure. Direct-sputtering ITO or IZO as the top electrode in the p-i-n device architecture can easily cause damage to the underlying perovskite layer due to the bombardment of high-energy particles during sputtering. To address this issue, the common approach is to use ALD to deposit a thin layer (or bilayer) of oxides, *e.g.*, SnO_2_ or SnO_2_/ZTO, before sputtering the TCO layer.^20^ The ALD-coated thin oxide layer works as a buffer or barrier layer with minimal light photon absorption and efficient electron extraction to protect the underlying perovskite layer from sputtering damage.^20^

To accommodate the fabrication process of perovskite subcells, the Si bottom cell with a flat, nontextured, front surface was initially used to demonstrate a 13.7%-efficient 2T perovskite/Si tandem solar cell in 2015.^129^ In this study, an n^++^/p^++^ Si tunnel junction was used to connect the Si bottom subcell to an n-i-p perovskite top subcell. An ITO-based interconnection layer was soon developed to improve the 2T perovskite/Si tandem performance to 19.9%, based on a flat Si heterojunction (SHJ) cell, and enabled by utilizing a low-temperature ALD-coated SnO_2_ ETL in the perovskite subcell.^130^ With a similar tandem structure, the 2T tandem efficiency was further pushed to 21.2% by using an antireflection foil to increase light harvesting and using IZO instead of ITO as the interconnection layer.^131^

In 2017, a p-i-n perovskite subcell structure was used in the perovskite/Si tandem based on a flat SHJ subcell.^20^ In this study, a low-temperature NiO_*x*_ HTL was used to accommodate the temperature constraint from the bottom SHJ subcell. To make a highly efficient semitransparent p-i-n perovskite subcell, an ALD-coated SnO_2_/ZTO bilayer structure was introduced prior to sputtering the ITO top electrode. This ALD oxide bilayer provides good protection of the underlying perovskite from sputtering damage. A certified 23.6% efficiency was reached at the 1 cm^2^ device level. This study marks a turning point in perovskite/Si 2T tandem development as the basic tandem architecture. In particular, the perovskite subcell structure reported in this study has been adopted by most groups to further advance perovskite/Si 2T tandem devices involving a Si bottom cell with a flat front surface.

With recent advances in wide-bandgap PSC development, perovskite/Si 2T tandem solar cells have been increased to ∼27% efficiency.^26,103^ In one study, 2D additive engineering with I^–^-SCN^–^-mixed PEA(I_0.25_SCN_0.75_) was used to improve charge transport in a 2D/3D perovskite structure, leading to a demonstration of a 1.68 eV (FA_0.65_MA_0.2_Cs_0.15_)Pb(I_0.8_Br_0.2_)_3_ PSC with 20.7% efficiency and less than 20% degradation after 1000 h of continuous illumination.^26^ In another study, adding a 3-mol % MAPbCl_3_ to the precursor of FA_0.78_Cs_0.22_Pb(I_0.85_Br_0.15_)_3_ resulted in the formation of 1.67 eV, triple-halides-based perovskite thin films with much improved structural properties, enhanced carrier lifetime/mobility, and reduced defect density.^103^ The corresponding semitransparent wide-bandgap PSC showed less than 4% degradation after 1000 h of maximum power point operation at 60 °C.

During the past 1–2 years, there has also been significant progress on developing perovskite/Si 2T tandem on Si bottom cells with a textured front surface. In general, Si cells with a textured surface can enhance light harvesting. However, the challenge to grow a perovskite subcell on a textured Si subcell is the shunting caused by the sharp and large texture features. Early efforts involved the use of nanocrystalline, Si-based tunnel junctions (*e.g.*, nc-Si:H[n^+^]/nc-Si:H[p^+^]), coupled with hybrid two-step sequential coating of a conformal perovskite layer.^49^ Two recent studies have separately demonstrated viable solution processing for preparing perovskite subcells on a textured Si surface.^24,32^ Both studies showed that preparing a micrometer-level-thick perovskite layer by either blade coating or spin coating, using a high concentration perovskite precursor together with Si subcells having reduced texture size, is a good strategy to improve the perovskite/Si tandem devices with a textured Si front surface.

Early in 2020, the efficiency of perovskite/Si 2T tandem solar cells reached a certified value of 29.15% with a ∼1 cm^2^ device area.^2^ This has significantly surpassed the best reported 4T perovskite/Si tandem with a 27.1% PCE.^23^ Although the details of the 29.15% 2T tandem device architecture are still undisclosed, this performance level provides confidence in perovskite/Si 2T tandem PV to reach above the 30% efficiency milestone in the near future, which, in turn, further justifies the value of pursuing perovskite tandem PV beyond the current single-junction limitation for commercial PV applications.

*Perovskite/CIGS Tandem*. The perovskite solar cell is a polycrystalline thin-film PV technology. CIGS is another well-established polycrystalline thin-film PV absorber with an optimum bandgap of approximately 1.1 eV and a certified PCE of 23.35% with *J*_sc_ near 40 mA/cm^2^.^2^ Thus, it is attractive to pair a CIGS cell with a ∼1.7 eV wide-bandgap PSC to form efficient and stable polycrystalline thin-film tandem solar cells. The performance potential is similar to that of the perovskite/Si tandem device.

Like the perovskite/Si tandem architecture, a TCO-based interconnection layer is often used to construct perovskite/CIGS 2T tandem solar cells. For example, an ITO layer was used as the interconnection layer in the early demonstration of a functional perovskite/CIGS 2T tandem device with 10.9% PCE.^46^ Owing to the polarity of the standard CIGS cell structure, a p-i-n perovskite subcell is always used for perovskite/CIGS 2T tandem devices. This allows direct utilization of p-i-n wide-bandgap PSC, including the top transparent electrode (*e.g.*, ALD-coated SnO_2_ and sputtered ITO or IZO), developed for perovskite/Si 2T tandem solar cells.

The surface roughness of CIGS cells normally ranges from dozens to hundreds of nanometers, which makes it hard to form a high-quality perovskite film on top. The rough surface of the CIGS subcell can also easily generate shunts. Thus, the large surface roughness of the CIGS subcell presents a challenge for developing a highly efficient perovskite/CIGS 2T tandem device, similar to the challenge encountered when preparing a perovskite subcell on a textured Si bottom subcell. To address this issue, several approaches have been explored with encouraging results. In one study,^25^ a thick ITO layer of about 300 nm was deposited on top of a CIGS subcell, followed by chemical/mechanical polishing to planarize the starting surface for perovskite deposition. With this approach, the effective surface roughness of the bottom CIGS subcell was reduced to a few tens of nanometers from the initial few hundred nanometers, enabling the demonstration of a certified 22.43%-efficient 2T tandem device with about 12% degradation over 500 h of continuous operation under 1-sun illumination.^25^ In another recent study, self-assembling monolayer (SAM) molecules were used directly on top of the AZO layer in a rough CIGS subcell. This SAM layer works as an effective HTL, enabling a 2T tandem device with a certified 23.26% efficiency and 1 cm^2^ active area.^36^ At present, the best perovskite/CIGS 2T tandem device has reached 24.2%; however, the detailed device information is yet unknown.^2^ Note that this 2T performance level is currently still behind the best reported 4T perovskite/CIGS tandem with a 25.9% PCE.^26^

*Perovskite/Perovskite Tandem*. Perovskite/perovskite tandem development is the most challenging among perovskite-based tandem technologies. (1) The biggest challenge is the development of highly efficient and stable low-bandgap PSCs. Sn/Pb alloying has become the standard way to prepare perovskites with a low bandgap of 1.2–1.3 eV for perovskite/perovskite tandem applications. However, it is difficult to form uniform, pinhole-free Sn-containing perovskite thin films because of rapid crystallization. Besides, Sn^2+^ is susceptible to oxidization, resulting in high defect densities. In contrast, both Si and CIGS solar cells are mature PV technologies and are ready for tandem integration with no need for significant further development. (2) The second challenge is the processing constraints during tandem integration. In comparison to Si and CIGS, perovskites often exhibit a much narrower tolerance to the processing environment during tandem integration, such as temperature, ambient gas environment, and processing solvent, to avoid damage to the underlying perovskite subcell. (3) Bandgap pairing for perovskite/perovskite tandem devices is more challenging than that for perovskite/Si and perovskite/CIGS tandem devices. For Si and CIGS, approximately 1.7 eV PSC is ideal for pairing for the optimum tandem performance. At present, the lowest achievable bandgap for Sn/Pb perovskites is near 1.2 eV,^27,28,132^ which would ideally require a wide bandgap near 1.8 eV for tandem devices.^55^ A wider bandgap requires more Br incorporation, which presents a challenge for the wide-bandgap PSC development, primarily associated with the *V*_oc_ loss and halide-phase segregation, as discussed in previous sections. Despite these unique challenges for perovskite/perovskite tandem development, various approaches have been explored in the past few years to address one or more of these challenges to steadily push forward the perovskite/perovskite tandem development.

In 2015, Heo and Im demonstrated a 2T all-perovskite tandem device by laminating two separate MAPbI_3_ and MAPbBr_3_ perovskite subcells with a 2 μm thick PTAA interconnection layer, producing a tandem PCE of only 10.4%.^133^ With significant advances on the interconnection layer and low-bandgap PSC development, 2T all-perovskite tandem performance has now reached a range of ∼23%–25% PCEs by multiple groups.^27,28,38,132^ The requirement of the interconnection layer for an all-perovskite tandem is similar to that for the transparent electrode used in the semitransparent wide-bandgap PSCs. As such, a combination of a thin oxide buffer layer (*e.g.*, SnO_2_, SnO_2_/ZTO, AZO, or MoO_*x*_) and a TCO layer (ITO or IZO) is frequently used as the interconnection structure.^28,29,38,53,134,135^ The TCO layer not only provides electrical connection between the top and bottom perovskite subcells; it also protects the underlying perovskite subcell from solvent damage during the preparation of another perovskite subcell on top. The TCO layer, normally ITO or IZO, is usually deposited by sputtering. The oxide buffer layer protects the underlying perovskite layer from possible TCO sputtering damage. In 2016, Eperon *et al*. used SnO_2_/ZTO/ITO as the interconnection layer to build a 17%-efficient perovskite/perovskite 2T tandem device.^134^ The adoption of a thin (∼2 nm) ZTO layer in the interconnection stack reduces the contact resistance connecting two subcells. In this early tandem demonstration, the ITO layer is about 100 nm thick to protect the bottom perovskite subcell. After this work, many follow-up studies have adopted a thick ITO layer (100 nm or thicker) to further improve the performance of the 2T all-perovskite tandem solar cells.^28,29,44,135^ In 2019, Tong *et al*. reported an additive-assisted growth approach to suppress defect formation in Sn/Pb perovskites, producing a high-quality Sn/Pb low-bandgap perovskite thin film with carrier lifetime over 1 μs, leading to demonstrations of >20%-efficient single-junction low-bandgap PSCs along with >23%-efficient 2T and >25%-efficient 4T all-perovskite tandem solar cells.^28^ In this study, the 2T tandem interconnection structure was adopted from a previous study by Zhao *et al*.,^29^ and it was also based on a thick ITO layer (∼120 nm), along with a thermally evaporated bilayer of 1 nm Ag and 3 nm MoO_*x*_—rather than a ALD-coated SnO_2_ layer—with Ag serving recombination sites and MoO_*x*_ as a barrier against ITO sputtering damage.

Although the thick ITO layer in the interconnection stack can provide good protection during subsequent perovskite subcell processing, it increases parasitic absorption and lateral conductivity, both of which are problematic for 2T tandem development. Palmstrom *et al*. recently showed that using a nucleation layer of poly(ethylenimine) ethoxylated (PEIE) prior to ALD coating the AZO oxide buffer layer can make the buffer layer dense and conformal, which is sufficient to function as a solvent barrier, enabling a coating of 5–10 nm thick IZO layer for efficient vertical charge conduction and minimum shunts from the two subcells.^38^ As a result, the 2T all-perovskite tandem solar cells reached 23.1% efficiency on a rigid glass and 21.3% efficiency on a flexible polyethylene napthalate (PEN).^38^ Lin *et al*. further eliminated TCO by using only 20 nm compact SnO_2_ and 1 nm Au in the interconnection structure.^27^ Using this simplified interconnection structure, along with a Sn-reduced precursor solution strategy, researchers demonstrated a PCE of 24.8% for a 0.049 cm^2^ 2T tandem device.^27^ Later, a certified efficiency of 24.2% was demonstrated for a 2T tandem with an area over 1 cm^2^ using surface-anchoring zwitterionic antioxidant.^136^ In these two studies by Tan *et al*., the wide-bandgap perovskite was based on Cs_0.2_FA_0.8_PbI_1.8_Br_1.2_; interestingly, a 6-precursor approach (based on mixing FAI, FABr, CsI, CsBr, PbI_2_, and PbBr_2_) was found to outperform a 4-precursor approach (based on mixing CsI, FAI, PbI_2_, and PbBr_2_) although the final perovskite composition is nominally the same. Along the direction of reducing the TCO layer thickness, Yu *et al*. recently showed a TCO-free interconnection scheme, where a bilayer of C60/SnO_1.76_ was used to directly connect two perovskite absorbers for constructing highly efficient and stable 2T tandem solar cells, with a PCE of 24.4% at 0.059 cm^2^ and 22.2% at 1.15 cm^2^, and with about 6% degradation after 1,000-h continuous 1-sun operation.^132^

*Other Emerging Perovskite-Based Tandem*. In addition to the commonly studied perovskite/Si, perovskite/CIGS, and perovskite/perovskite tandem devices, there are a few other emerging perovskite-based tandem configurations, such as perovskite/CdTe, perovskite/organic semiconductor, and perovskite/colloidal quantum dot tandem cells.

CdTe is currently the most successful thin-film PV technology. However, CdTe has a bandgap of ∼1.45 eV, which is not optimal to tandem with the regular wide-bandgap perovskites. A recent study calculated the detailed balance efficiency limit of perovskite/CdTe tandems and found that the maximum PCE for 4T perovskite/CdTe tandems is 39.5% with 2.11 eV perovskite as the top cells, while the maximum PCE is 39.3% for 2T configuration with a 1.98 eV perovskite top subcell.^137^ According to this calculation, the theoretical efficiency is significantly lower compared with that for perovskite/Si and perovskite/CIGS tandem configurations. On the other hand, PSCs based on perovskites with a bandgap around 2.0–2.1 eV currently exhibit very poor efficiency, especially for the *V*_oc_ loss, and they may also suffer from poor stability. In the case where CdTe is used as the top cell, the bottom cell needs to have an ideal bandgap below 1 eV,^138^ which would require a different low-bandgap absorber technology than perovskites.

Organic photovoltaics (OPV) have some advantages to tandem with PSCs owing to the flexibility in bandgap tuning as well as similar device form factors and processing methodologies.^139−141^ In 2015, Chen *et al*. employed MAPbI_3_ and PBSeDTEG8:PCBM as the top and bottom subcells, respectively, to fabricate 2T perovskite/organic tandem solar cells and achieved an efficiency of 10.2%.^140^ Recently, with development of wide-bandgap PSCs and improved OPV efficiency, the perovskite/organic tandem devices have reached above 20%. Xu *et al*. used the PBDBT-2F:Y6:PC71BM (*E*_g_ ≈ 1.41 eV) as the bottom cell and FA_0.8_MA_0.02_Cs_0.18_PbI_1.8_Br_1.2_ (*E*_g_ ≈ 1.77 eV) as the top cell to fabricate 2T perovskite/organic tandem solar cells with 20.6% PCE and good reproducibility.^141^ In the same study, they also used a semiempirical device model to predict a practical efficiency of >30% is possible with further development of low-bandgap OPV and wide-bandgap PSCs.

Chalcogenide colloidal quantum dots (CQDs), such as PbS and PbSe, have tunable bandgaps with strong absorption in the infrared region. Thus, CQD-based solar cells represent another type of low-bandgap absorber technology that can be paired with wide-bandgap PSCs for tandem applications. A recent study demonstrated a functional tandem device based on MAPbI_3_/PbS CQD with a PCE of 11.03%.^142^ Although the PCE of the perovskite/CQD tandem is low at present, a recent modeling study suggests a potential PCE of 29.7% is reachable by integrating the state-of-the-art wide-bandgap PSCs and CQD solar cells.^143^

In summary, the rapid progress of efficiency and stability, along with promising scalability demonstrations, has placed perovskite solar cells at an unprecedented place for future potential large-scale deployment of perovskite PV. Owing to the unique, demonstrated capability of perovskite-based wide-bandgap solar cells, another attractive and practical value of PSCs may come first by enabling stable and highly efficient tandem devices consisting of wide-bandgap PSCs and other mature PV technologies, such as Si and CIGS. This can help PSCs to penetrate the extremely competitive PV market without directly competing with other well-established commercial PV technologies. Efficiency and stability are always the two core research topics. For wide-bandgap PSCs, the main limitation of efficiency and stability are the larger *V*_oc_-loss with the increased bandgap and halide-related phase-segregation under illumination. To solve these two issues, more research efforts are needed to focus on improving the morphology of perovskite films, passivating traps in the bulk and/or at the surface of perovskite grains, and adjusting the interfacial electronic properties. In addition, developing alternative wide-bandgap perovskites via all-inorganic or nanostructured perovskites could avoid the issue of using mixed halide and mitigate halide-related *V*_oc_ loss and phase segregation challenges. At present, the efficiency of perovskite/Si tandem solar cells at a laboratory scale is already very high to justify the benefit of pursuing the tandem approach for the Si PV industry. For market adoption, further research should focus on stability and scalability using deposition methods with high throughput, high-device yield, and suitability for large-area tandem device fabrication. All-perovskite tandem solar cells not only hold potential for low-cost, lightweight, and high-power density applications, but they are also compatible with rapid manufacturing by roll-to-roll or sheet-to-sheet printing. Despite progress of all-perovskite tandem resulting from recent breakthroughs on low-bandgap Sn–Pb PSC development, all-perovskite tandem is still at its early stage. There is still much room left toward realizing high-efficiency and stable tandem PSCs. Significant efforts are needed to further optimize wide-bandgap PSCs.
